# Differential Impact of Living Arrangement on Dietary Quality between Older Men and Women

**DOI:** 10.1093/geroni/igaf122.3569

**Published:** 2025-12-31

**Authors:** Qun Le, Elizabeth Procter-Gray, Lingming Chen, Danielle LoPilato, Kevin Kane, Sabrina E Noel, Wenjun Li

**Affiliations:** University of Massachusetts Lowell, Lowell, Massachusetts, United States; University of Massachusetts Lowell, Lowell, Massachusetts, United States; University of Massachusetts Lowell, Lowell, Massachusetts, United States; University of Massachusetts Lowell, Lowell, Massachusetts, United States; University of Massachusetts Lowell, Lowell, Massachusetts, United States; University of Massachusetts Lowell, Lowell, Massachusetts, United States; University of Massachusetts Lowell, Lowell, Massachusetts, United States

## Abstract

Consuming a healthy diet is essential for healthy aging and longevity. Due to health conditions, living environment, and other factors, older adults are at increased risk of malnutrition. Our study aims to explore association between living arrangement and diet quality, and how gender may modify this association. The Healthy Aging and Neighborhood Study (2021-2025) collected data from 614 community-dwelling people aged 65 years and older living in Central and Northeastern Massachusetts. Living arrangement was measured as living alone vs. living with another adult(s). Diet was evaluated via the Dietary Screening Tool (range 0-100) with a higher score indicating higher diet quality. Generalized linear regression models assessed associations between living arrangement and dietary quality overall, and stratified by gender, adjusting for socio-demographic information including age, race, education, income, and activity-specific balance confidence. Participants were an average age of 74.6 (±6.2) y, 57.7% were women, and 56.0% Non-Hispanic White, 32.9% Asian, and 8.6% Hispanic. The mean diet quality score was 67.3 (±12.5). After adjustment, women had higher dietary quality than men (b (SE) = 3.96 (1.15)). Living alone had a differential impact on dietary quality between men and women. Among men, the 16.9% who lived alone had better diets (b (SE) = 4.81 (1.97)). However, among women, those who lived alone (37.3%) had lower dietary quality (b (SE) = -5.93 (2.29)). In conclusion, living status may differentially impact of dietary quality for men and women. Future studies should explore the underlying mechanism of such gender differences, and provide gender-specific effective interventions.

